# Tryptophan-5-HT pathway disorder was uncovered in the olfactory bulb of a depression mice model by metabolomic analysis

**DOI:** 10.3389/fnmol.2022.965697

**Published:** 2022-10-10

**Authors:** Guanghui Chen, Siqi Zhou, Qiang Chen, Mengmeng Liu, Meixue Dong, Jiabao Hou, Benhong Zhou

**Affiliations:** ^1^Department of Pharmacy, Renmin Hospital of Wuhan University, Wuhan, China; ^2^Department of Orthopedics, Renmin Hospital of Wuhan University, Wuhan, China; ^3^Department of Pharmacy, Liyuan Hospital, Tongji Medical College, Huazhong University of Science and Technology, Wuhan, China; ^4^Department of Neurology, Renmin Hospital of Wuhan University, Wuhan, China; ^5^Department of Anesthesiology, Renmin Hospital of Wuhan University, Wuhan, China

**Keywords:** major depression, metabolomic, olfactory bulb, tryptophan hydroxylase, chronic mild stress

## Abstract

Major depression (MD) is a severe mental illness that creates a heavy social burden, and the potential molecular mechanisms remain largely unknown. Lots of research demonstrate that the olfactory bulb is associated with MD. Recently, gas chromatography-mass spectrometry-based metabolomic studies on depressive rats indicated that metabolisms of purine and lipids were disordered in the olfactory bulb. With various physicochemical properties and extensive concentration ranges, a single analytical technique could not completely cover all metabolites, hence it is necessary to adopt another metabolomic technique to seek new biomarkers or molecular mechanisms for depression. Therefore, we adopted a liquid chromatography-mass spectrometry metabonomic technique in the chronic mild stress (CMS) model to investigate significant metabolic changes in the olfactory bulb of the mice. We discovered and identified 16 differential metabolites in the olfactory bulb of the CMS treatments. *Metabolic pathway analysis by MetaboAnalyst 5.0* was generated according to the differential metabolites, which indicated that the tryptophan metabolism pathway was the core pathogenesis in the olfactory bulb of the CMS depression model. Further, the expressions of tryptophan hydroxylase (TpH) and aromatic amino acid decarboxylase (AAAD) were detected by western blotting and immunofluorescence staining. The expression of TpH was increased after CMS treatment, and the level of AAAD was unaltered. These results revealed that abnormal metabolism of the tryptophan pathway in the olfactory bulb mediated the occurrence of MD.

## Introduction

Major depression (MD) is a severe mental illness that creates a heavy social burden. The disease influences exceeding 300 million people yearly, which is an important reason for disability worldwide (Hamel et al., 2019). The human brain is so complicated that depressive pathogenesis is still largely unknown, although much effort has been made (Robinson, 2018). Current antidepressant therapy carries out lots of side effects in most depressed patients (Cipriani et al., 2018). Therefore, it is emergent to find a new way to study the pathogenesis of depression and seek new targets for antidepressants.

Metabolomics employs liquid chromatography-mass spectrometry (LC-MS), gas chromatography (GC)-MS, and so on to analyze micromolecule of biological samples qualitatively and quantitatively for exploring the pathophysiological mechanism of life process (Ding and Mohan, 2016; Wishart, 2016). Metabolomics has been widely used to study the pathogenesis of depression (Ling-Hu et al., 2021; Pu et al., 2021). An increasing body of evidence suggests that olfactory bulb dysfunction is closely associated with depression. The olfactory bulb volume and olfactory sensitivity in MD patients are reduced (Negoias et al., 2010). Olfactory bulb volume could predict the therapeutic outcome of MD patients (Negoias et al., 2016), and the lowered olfactory sensitivity could partly be predicted by high depression scores (Pause et al., 2001). The reduced neurogenesis and olfactory receptor neurons, and increased apoptosis in the olfactory bulb were discovered in the rodent model of depression (Yang et al., 2011a,b; Li et al., 2015; Cheng et al., 2016). Recently, a GC-MS-based metabolomic study on the olfactory bulb of depressive rats indicates that purine and lipid metabolism are disordered (He et al., 2020). As various physicochemical properties and extensive concentration range, a single analytical technique could not completely cover all metabolites (Williams et al., 2006), so it is necessary to adopt other metabolomic techniques to seek new biomarkers or molecular mechanisms for depression. This complementation was vital to seek biomarkers and study pathogenesis in MD (Zheng et al., 2013a,b).

Therefore, based on previous GC-MS research, we adopted LC-MS metabonomic method to explore meaningful metabolic changes in the olfactory bulb of chronic mild stress (CMS) depression mice model (Huang et al., 2020). Further, the probable abnormal metabolism of the tryptophan pathway was confirmed. The main purpose of this research is to discover several novel metabolic changes in the olfactory bulb of depression model mice and search for new mechanisms and therapeutic targets for depression.

## Materials and methods

### Animals

Thirty C57BL/6J mice (male; weight, about 22 g; age: 8 weeks) were obtained from Beijing Vital River Laboratory Animal Technology Co., Ltd. The mice were kept in a standard environment for feeding. Before the experiment begins, the mice were fed adaptively for 1 week. Then, the mice were weighed and randomly divided into the CMS group (*n* = 15) and Control (CON) group (*n* = 15). The schedule of CMS was according to the previous research with few alterations (Crowley et al., 2004; Xie et al., 2022). All experimental procedures were ratified by the Institutional Animal Care and Use Committee of Wuhan University (IACUC Issue No. WDRM20210123) and carried out based on the Declaration of the Health Guide for Care and Use of Laboratory Animals formulated by Wuhan University.

### CMS treatment

**Figure 1:**
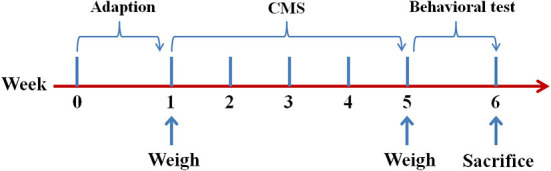
Time schedule for the CMS procedure and behavior tests. CMS, chronic mild stress.

As shown in Figure 1, the mice were exposed to CMS for 4 weeks before the behavioral tests. Stressors mainly include food deprivation (24 h), water deprivation (1 day), wet bedding (1 day), inversion of dark/light cycle (12 h), cage tilting (45°, 1 day), tail pinching (2 min), and shaking cage (horizontal, 5 min). The CMS mice were given a stressor every day randomly and 2 days in a row without the same stressor.

### Forced swimming test (FST)

FST was conducted based on previous research (Xie et al., 2022). In general, the mice were separately placed in glass cylinders (height, 25 cm; diameter, 12 cm) full of 18 cm of water (25°C) for testing. The mice were put in the cylinder for 6 min, and the duration of immobility was calculated in the last 4 min. Immobility was defined as floating or remaining motionless, which means the absence of all movement except motions required to maintain the head above the water.

### Tail suspension test (TST)

The TST was conducted to assess behavioral despair of mice as previously described (Crowley et al., 2004). The mouse's tail was held in place with tape and its head 30 cm above the ground. The immobile time was recorded by video system in the 6 min experimental process. The state that the mice were motionless without any struggle was defined as immobility.

### Open field test (OFT)

Locomotor activity and exploratory behavior were evaluated by OFT based on previous research (Xie et al., 2022). In general, the mice were separately placed in the apparatus (transparent plastic square: 45 cm × 45 cm). The mice were put in the open field center and recorded for 5 min after the beginning of the experiment. The whole activity of the animal was monitored by a video system. The video system was used to calculate the total distance and rearing frequency.

### Sample collection and preparation

Isoflurane was used to anesthetize mice when the behavioral experiments were finished. Once the righting reflexes of mice disappeared, they were rapidly sacrificed and the olfactory bulb was collected.

### LC/MS analysis

We adopted an LC/MS metabolomic method to conduct this research and the specific procedure was described in previous studies (Dong et al., 2018; Chen et al., 2019; Xie et al., 2022). The detailed information was described in the Supplementary materials.

### Histological and immunofluorescence analysis

The olfactory bulbs were embedded in paraffin after 4% paraformaldehyde solution overnight.

Hematoxylin–eosin was used to stain the slices. Every 5th section of them was preserved and taken photos by an Olympus light microscope. The maximal cross-sectional areas and diameter of the olfactory bulb were calculated, which were estimated by the Photo Imaging System according to the sections.

For immunofluorescence analysis, the olfactory bulb slices were dewaxed and potched by phosphate buffer saline (PBS). The olfactory bulb tissue slices were blocked for 30 min in bovine serum albumin (BSA) (3%) and fetal bovine serum (2 in 0.2% Triton X-100/PBS) after antigen retrieval, and incubated with a primary antibody (4°C, overnight) for tryptophan hydroxylase (TpH) (abcam 52,954; dilution, 1:300) and aromatic amino acid decarboxylase (AAAD) (abcam 142,497; dilution, 1:500). Then, the slices were incubated in a biotinylated secondary antibody and an avidin-biotinylated horseradish peroxidase complex solution, ordinally. 4,6-diamidino-2-phenylindole (DAPI) (1:500, 5 min) was used to stain nuclei. Negative controls were conducted by omitting the primary antibody and presented with negligible background fluorescence. At last, peroxidase activity was detected by a diaminobenzidine staining kit and observed in the Photo Imaging System (five fields of different samples).

### Western blot assay

BCA Protein Assay Kit was adopted to detect the total protein concentration of the olfactory bulb. Then, the proteins were separated by sodium dodecyl sulfate–polyacrylamide gel electrophoresis and transferred to polyvinylidene fluoride membranes, furtherly. The membranes were incubated with the primary antibodies (overnight, 4°C): TpH (dilution, 1:2,000), AAAD (dilution, 1:1,500), and glyceraldehyde 3-phosphate dehydrogenase (GAPDH) (dilution, 1:2,000). Then, the membranes were washed by TBST (3 times/10 min) and reacted with horseradish peroxidase-conjugated secondary antibodies (1:15,000) (room temperature, 1 h). The ChemiDoc XRS + System was used to detect the immunoreactive bands. The protein expression was normalized by GAPDH.

### Statistical analysis

SPSS 18.0 was adopted to analyze the data. Means ± S.E.M. was used to convey quantitative data. Student's *t*-tests were adopted to process the data of behavior test and protein expression, and *P* < 0.05 was regarded as statistical significance. *MetaboAnalyst 5.0* was adopted to achieve the significant pathways for capturing the disturbed metabolic pathway (*P* < 0.05).

## Results

### CMS model establishments

**Figure 2:**
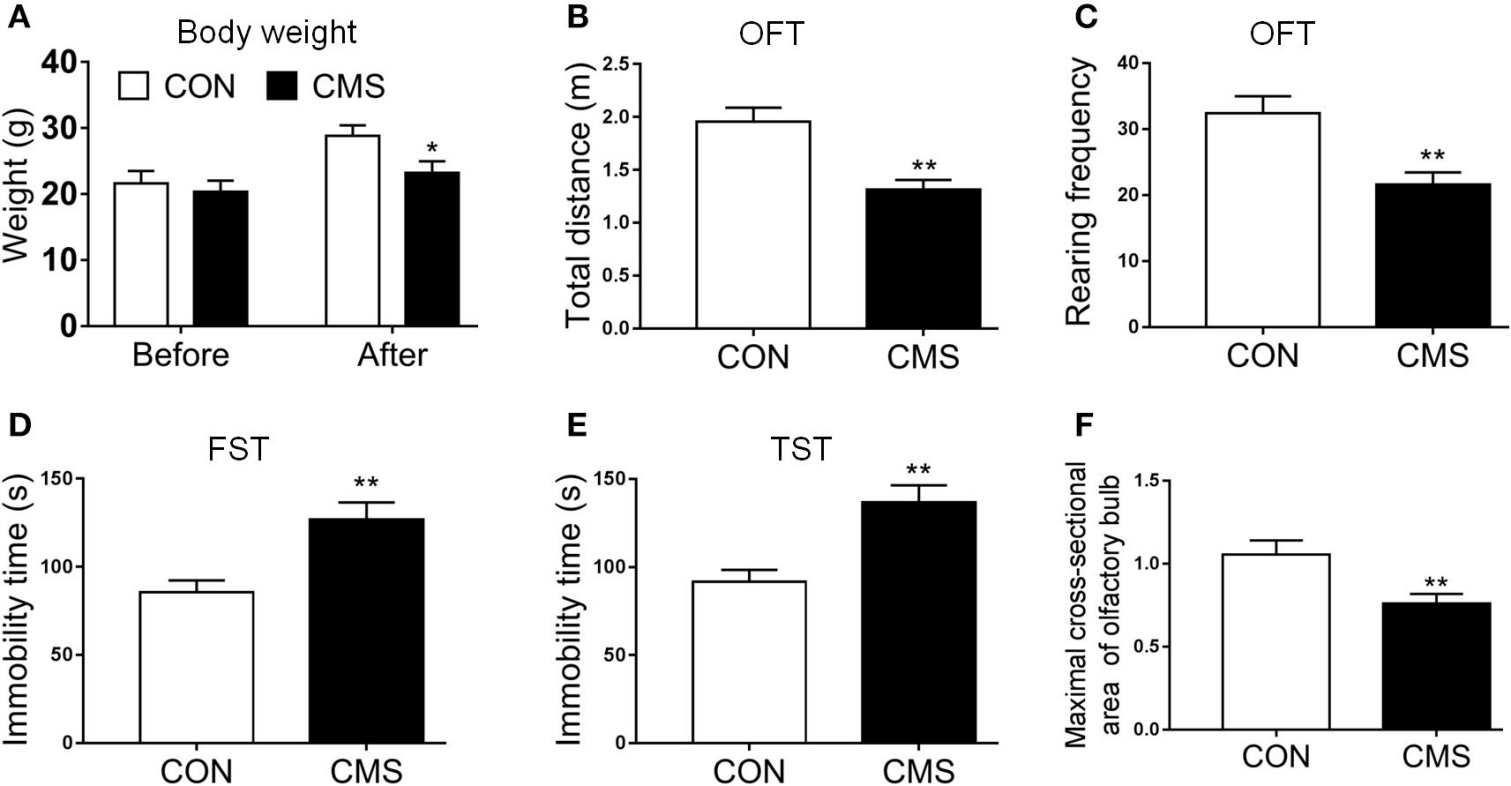
Evaluating the results of the CMS depression model. **(A)** Body weight before and after the CMS; **(B,C)** Total distance of central zone and rearing frequency in OFT; **(D,E)** The immobile time in FST and TST; **(F)** Olfactory bulb maximal cross-sectional area and diameter. Data are expressed as means ± S.E.M. *P < 0.05, **P < 0.01 as compared with control group. CMS, chronic mild stress; OFT, open field test; TST, tail suspension test; FST, forced swimming test. *n* = 15 for weight and behavior test and *n* = 3 for olfactory bulb maximal cross-sectional areas and diameter.

We employed the indicators (body weight, OFT, FST, and TST) to evaluate the quality of the CMS model. The body weight of CMS mice was less than CON mice after CMS, while no significant differences were discovered between them at baseline [Figure 2A, *t*(10) = 1.56, *P* < 0.05]. In OFT, the number of locomotor and rearing activities in mice subjected to CMS for 4 weeks was decreased significantly compared to CON mice [Figures 2B,C, *t*(10) = 5.81, *P* < 0.01, *t*(10) = 3.26, *P* < 0.01]. Regarding FST and TST, the immobility time in CMS mice was increased compared to CON mice [Figures 2D,E, *t*(10) = 3.28, *P* < 0.01, *t*(10) = 4.56, *P* < 0.01]. In addition, we found the maximum cross-section area of the olfactory bulb in CMS mice was reduced compared to CON mice [Figure 2F, *t*(4) = 2.89, *P* < 0.01].

### Metabolomic analysis and differential metabolite identification

**Figure 3:**
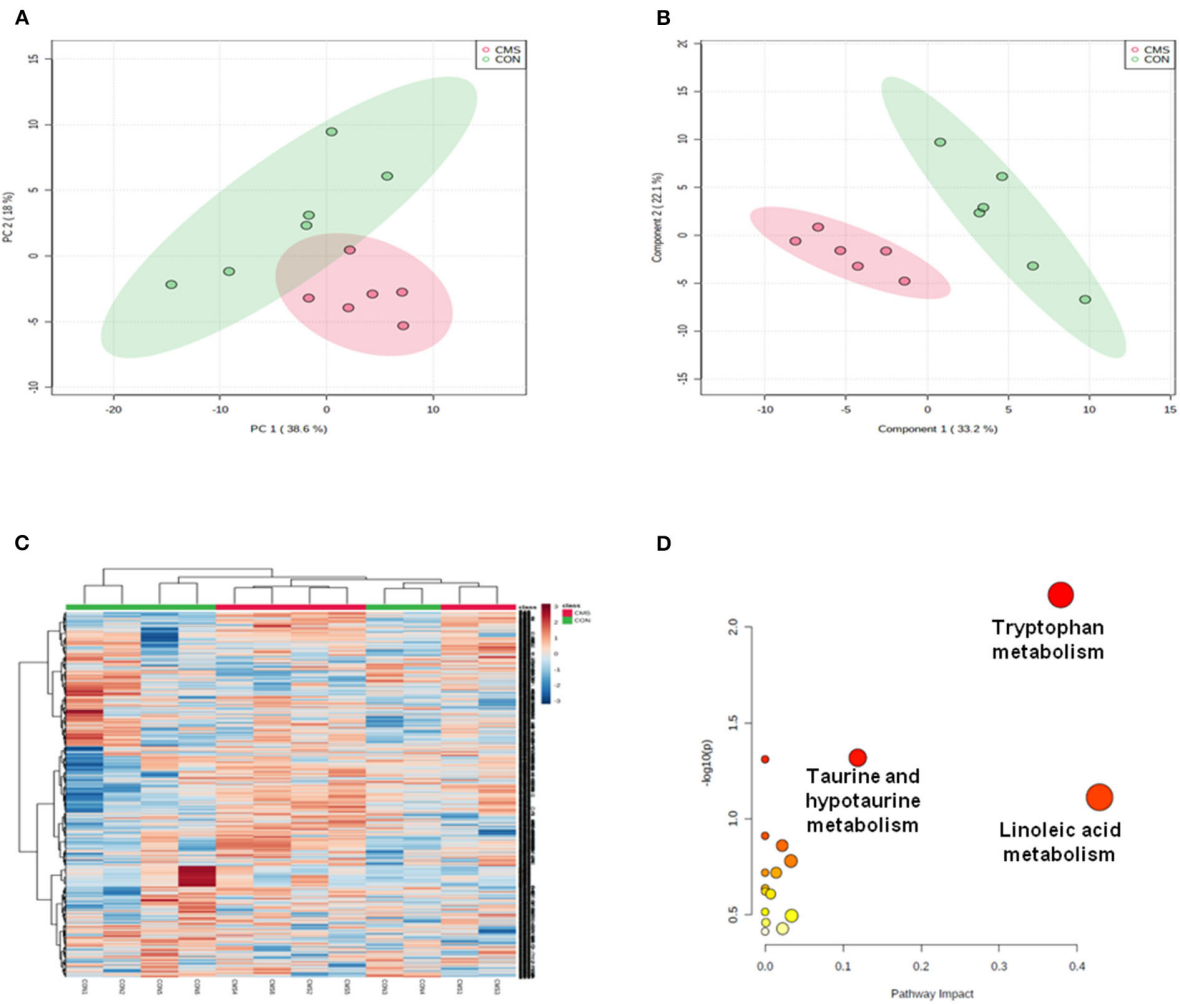
Multivariate statistical analysis and metabolic pathway analysis. **(A)** Principal component analysis (PCA) scores plot derived from LC/MS of the chronic mild stress (CMS) and healthy control (CON) groups. **(B)** Pair-wise orthogonal projections to latent structures discriminant (OPLS–DA) scores plot derived from LC/MS of the CMS and CON groups. **(C)** Clustering analysis for the differential metabolites. **(D)** Pathway analysis; a, tryptophan metabolism; b: linoleic acid metabolism; c: taurine and hypotaurine metabolism. *n* = 6.

The data acquired through LC-MS were subjected to multivariate analyses (including negative ionization and positive ionization). The clear differences were shown by PCA scores plot in CMS and CON groups (Figure 3A). Further, statistical difference was indicated by OPLS–DA score plots between CMS mice and CON mice (*R*^2^*X* = 0.866, *R*^2^*Y* = 0.821, and *Q*^2^ = 0.913) (Figure 3B). At last, a total of 16 differential metabolites were identified between them (Table 1). CMS mice were characterized by an increased level of uric acid (UA), methacholine, sorbitol, inosine, taurine, acetone, ribitol, and metanephrine compared with controls, as well as a reduced level of phosphatidylcholine (PC) O-34:2, PC[20:0/22:1(13Z)], tryptophan, 5-hydroxytryptamine (5-HT), 5-hydroxy-L-tryptophan (5-HTP), fructose-6-phosphate, spermidine, and glucose.

**Table 1:** List of differential metabolites between CMS group and CON group.

**Number**	**Differential metabolites**	**VIP**	**Abundance**		***t*(10)**	***P***
			**CMS**	**CON**		
1	Uric acid	2.06	3.54 ± 0.18	2.06 ± 0.16	4.91	**
2	Methacholine	1.87	8.64 ± 0.63	6.67 ± 0.87	1.53	*
3	Sorbitol	1.97	2.75 ± 0.15	2.21 ± 0.15	2.69	**
4	Inosine	2.08	2.06 ± 0.16	1.39 ± 0.087	6.45	**
5	Taurine	1.74	10.75 ± 1.38	8.76 ± 0.83	1.61	*
6	Acetone	2.53	15.34 ± 1.37	4.06 ± 0.36	5.52	**
7	Ribitol	1.97	0.46 ± 0.024	0.22 ± 0.015	3.56	**
8	Metanephrine	1.65	1.21 ± 0.13	0.24 ± 0.018	3.12	**
9	PC O-34:2	2.08	21.87 ± 1.26	27.6 ± 1.97	2.68	**
10	PC(20:0/22:1(13Z))	1.92	23.34 ± 1.48	33.35 ± 2.86	2.08	**
11	Tryptophan	1.67	1.57 ± 0.12	2.88 ± 0.23	4.36	**
12	5-HT	2.53	4.08 ± 0.58	5.7 ± 0.61	1.39	*
13	5-HTP	1.75	26.98 ± 1.56	33.24 ± 2.86	7.67	**
14	Fructose-6-phosphate	2.54	0.21 ± 0.017	1.05 ± 0.13	6.28	**
15	Spermidine	2.17	0.68 ± 0.033	1.28 ± 0.11	3.86	**
16	Glucose	1.79	4.98 ± 0.69	7.27 ± 0.86	1.26	*

CMS, chronic mild stress; CON, control; PC, phosphatidylcholine; 5-HT, 5-hydroxytryptamine; 5-HTP, 5-hydroxy-L-tryptophan; *P < 0.05; **P < 0.01.

### Metabolic pathway analysis by metabo analyst 5.0 and verification

**Figure 4:**
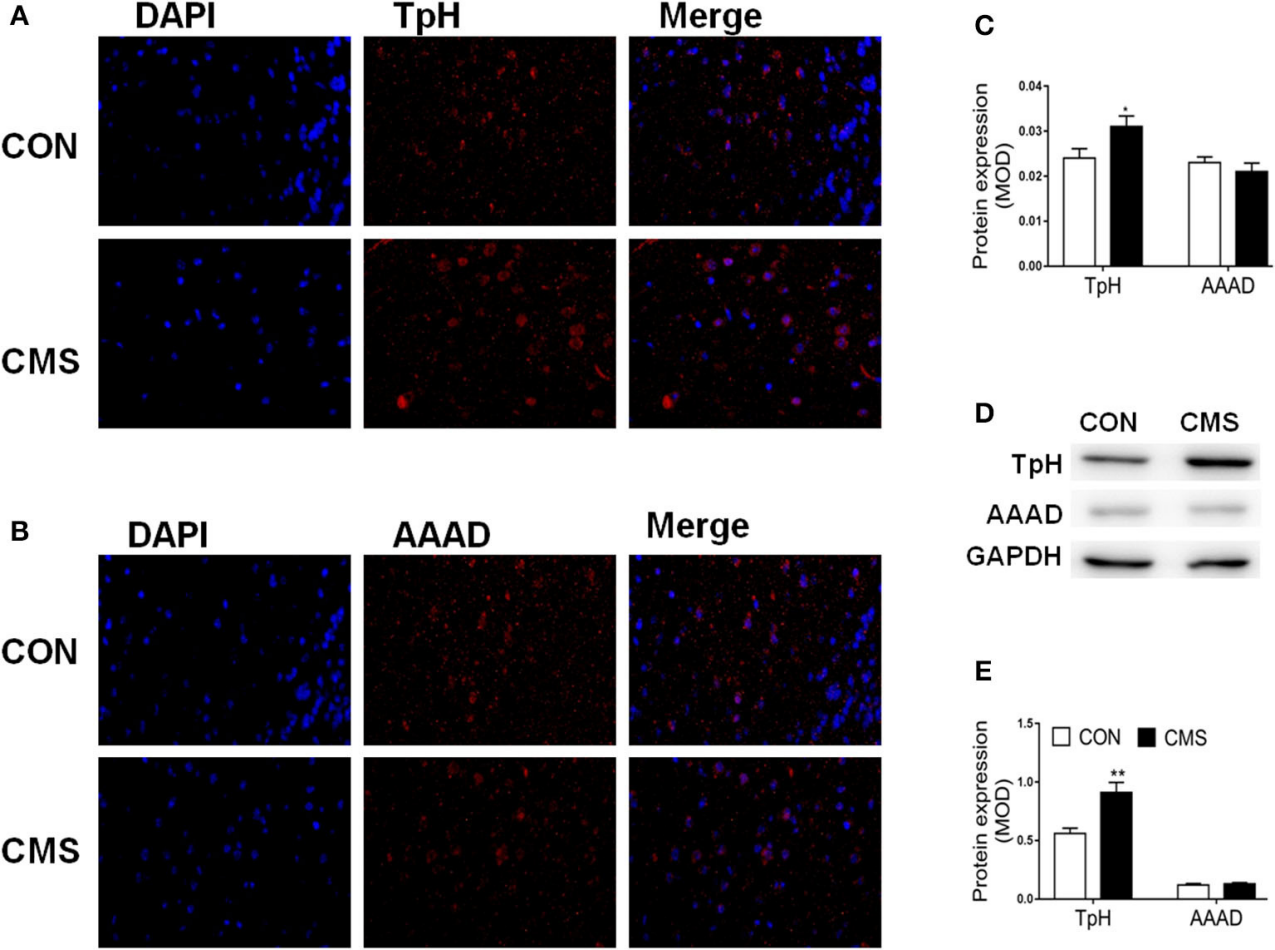
The protein expression of tryptophan hydroxylase (TpH) and aromatic amino acid decarboxylase (AAAD) in the olfactory bulb of chronic mild stress mice. **(A–C)** The protein level of TpH and AAAD by immunofluorescence; **(D, E)** The protein level of TpH and AAAD by Western blot. *n* = 3.

We used *Metabo Analyst 5.0* to analyze the metabolic profiling and found that all metabolites of CMS mice and CON mice were shown in the hierarchical clustering heatmap (Figure 3C). Then, we used *Metabo Analyst 5.0* to perform metabolic pathway analysis according to the 16 differential metabolites. Three differentially metabolic pathways are discovered (Figure 3D, *P* < 0.05): (1) Tryptophan metabolism (impact = 0.38, *P* < 0.05), (2) Linoleic acid metabolism (impact = 0.43, *P* < 0.05), and (3) Taurine and hypotaurine metabolism (impact = 0.19, *P* < 0.05). The results indicated that the tryptophan metabolism pathway of the olfactory bulb was involved in the underlying pathogenesis of depression. In general, tryptophan formates indoleamine, which produces 5-HTP by TpH and 5-HT by AAAD (Maffei, 2020). At last, we adopted immunofluorescence and western blot to detect the expressions of TpH and by AAAD for verification. We discovered that the protein expression of TpH was increased in the olfactory bulb of CMS mice compared with CON mice, while the protein expression of AAAD was unchanged [Figures 4A–E, *t*(4) = 1.55, *P* < 0.05, *t*(4) = 4.69, *P* < 0.01].

## Discussion

### The main findings of this study

Lots of evidence suggest that olfactory bulb dysfunction is associated with depression. Olfactory bulb volume in MD patient was reduced and recovered after antidepressant treatment (Negoias et al., 2010, 2016). In this research, we first discovered the maximum cross-section area of the olfactory bulb in CMS mice was reduced. Then, we used LC/MS (metabolomics techniques) to study the metabolic alterations of the olfactory bulb. We discovered 16 metabolites categorized into three influenced pathways by OPLS-DA analysis and *MetaboAnalyst*. The metabolic disturbance of olfactory (including tryptophan metabolism, linoleic acid metabolism, and taurine and hypotaurine metabolism) was regarded as the main pathway that mediated the onset of depression. Eight differential metabolites were increased and 8 were decreased, which may be therapeutic targets of depression. The previous metabolomic study of the olfactory bulb by GC-MS found that 19 metabolites were altered in CMS rats compared to CON rats (He et al., 2020). These metabolites were highly associated with the disturbances of purine and lipids metabolism.

Three influenced metabolic pathways were relevant to differential metabolites. Our results indicated that disturbance of these metabolites may refer to promoting depressive-like phenotypes and olfactory bulb disorder in CMS mice. The abnormal tryptophan metabolism has been reported to associate with MD and depression model (Wang et al., 2021; Tian et al., 2022). What's more, we first found that linoleic acid metabolism and taurine and hypotaurine metabolism mediated the onset of depression.

### Tryptophan-5-HT pathway

**Figure 5:**
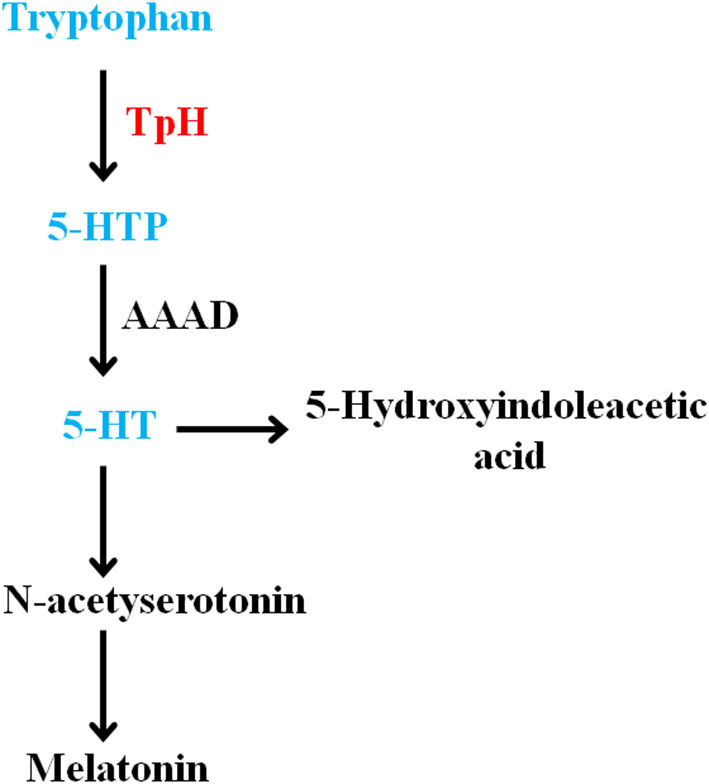
Summary of the olfactory bulb metabolites involved in the disturbance of tryptophan-5-HT pathway in chronic mild stress mice. 5-HT, 5-hydroxytryptamine; 5-HTP, 5-hydroxy-L-tryptophan.

Tryptophan is an essential acid used for synthesizing proteins. It is also the precursor of the neurotransmitter serotonin (Slominski et al., 2002). The monoamine neurotransmitters (including 5-HT) can modulate mood, cognition, and so on (Young, 2007). In general, tryptophan formates indoleamine, which produces 5-hydroxytryptophan by TpH and 5-HT by AAAD (Maffei, 2020). The detailed metabolic steps of the tryptophan-5-HT pathway are summarized in Figure 5.

The metabolic disturbance of tryptophan and 5-HT was vital for MD. Plasma tryptophan levels in depression (including MD, suicidal MD patients, and depression model rats) were reduced (Messaoud et al., 2019, 2021; Li et al., 2020), and reversed after antidepressant treatment (Ciocan et al., 2021). In addition, tryptophan supplementation could improve symptoms of depression in MD patients (Gonzalez et al., 2021). The decreased 5-HT transporter availability was observed in depressed patients (Staley et al., 2006). The plasma 5-HT level was reduced in depression patients and increased after antidepressant administration (Blardi et al., 2002), and the plasma 5-HT level of responders was increased compared to nonresponders (Celada et al., 1992). The 5-HT level of the prefrontal cortex and hippocampus was downregulated in the acute and chronic stress-induced depression model (Jia et al., 2017; Zhang et al., 2018). In this study, we discovered that the levels of metabolites (including tryptophan, 5-HTP, and 5-HT) were reduced in the olfactory bulb of CMS mice. Then, we discovered that the protein expression of TpH was increased and AAAD was unchanged in the olfactory bulb of CMS mice. These results suggested that TpH may be an intervention target for olfactory bulb metabolism abnormality mediating depression.

### PC

PC, a kind of phospholipids, is the main component of biomembrane and synthesizes choline. Clinical research indicated that plasma PC concentrations of depression patients were associated with the severity of depression (Demirkan et al., 2013). Plasma PC levels showed a positive correlation with depression status in postmenopausal women (Huang et al., 2021). Serum PC in the olfactory bulbectomy-induced depression model was increased, (Yan et al., 2021) while some PCs [PC (32:1), PC (37:4) and its like] in plasma of depressed rats were reduced (Chen et al., 2014). These differences may be due to the difference between the depression model and depressive phenotype. PC supplementation efficiently reversed the disorder of hippocampal neurogenesis by inhibiting circulating TNF-α levels (Tokés et al., 2011). The neuroplasticity of neural stem cells was damaged by inflammatory stress and could be restored by PC (Magaquian et al., 2021). The neurogenesis of the olfactory bulb was reduced (Yang et al., 2011a). Therefore, the reduced PC level of olfactory bulb mediated neurogenesis disorder in CMS mice.

### UA

The levels of serum and plasma UA in patients with depression and CMS rats were lower than in control, which was a depression biomarker (Peng et al., 2016; Xiong et al., 2016; Meng et al., 2020; Yuan et al., 2021; Ceresa et al., 2022). Plasma UA-adjusted mean levels were lower in current major depressive disorder compared to remitted disorders and controls (Black et al., 2018). The plasma UA levels showed a negative correlation with the risk of depression in patients and antidepressant use (Wium-Andersen et al., 2017). The plasma UA levels of comorbid unipolar depression were increased compared with the unipolar depression and healthy control (Ozten et al., 2015). The research indicates that increased serum UA levels in depressed patients have a positive correlation with hypomanic episodes or subsequent manic (Dos Santos Oliveira et al., 2019). The rates of hyperuricemia and serum UA levels in bipolar were significantly increased compared with the control (Albert et al., 2015). Therefore, the reduced UA level of the olfactory bulb may be a biomarker for CMS mice.

## Conclusion

We employed LC/MS metabolomics techniques to achieve metabolic profiling in the olfactory bulb of CMS mice. We discovered that tryptophan-5-HT pathway metabolism was disordered in the olfactory bulb of depression mice and TpH may be an intervention and treatment target for depression. Futher, we first discovered that linoleic acid metabolism and taurine and hypotaurine metabolism mediated the onset of depression.

## Data availability statement

The raw data supporting the conclusions of this article will be made available by the authors, without undue reservation.

## Ethics statement

The animal study was reviewed and approved by the Institutional Animal Care and Use Committee of Wuhan University. Written informed consent was obtained from the owners for the participation of their animals in this study.

## Author contributions

BZ performed the research. QC and SZ designed the research study. GC, ML, and MD analyzed the data and wrote the article. GC and JH revised the article. All authors contributed to the article and approved the submitted version.

## Funding

This research was funded by the National Natural Science Foundation of China (No. 82104312, No. 82001119), the National Natural Science Foundation of Hubei Province (No. 2020CFB258), and the Fundamental Research Funds for the Central Universities (No. 2042020kf0044).

## Conflict of interest

The authors declare that the research was conducted in the absence of any commercial or financial relationships that could be construed as a potential conflict of interest. The reviewer PZ declared a shared affiliation with the author QC to the handling editor at the time of review.

## Publisher's note

All claims expressed in this article are solely those of the authors and do not necessarily represent those of their affiliated organizations, or those of the publisher, the editors and the reviewers. Any product that may be evaluated in this article, or claim that may be made by its manufacturer, is not guaranteed or endorsed by the publisher.
