# Supplementary material for: Tryptophan-5-HT pathway disorder was uncovered in the olfactory bulb of a depression mice model by metabolomic analysis
Source: Front Mol Neurosci. 2022 Oct 10;15:965697. doi: 10.3389/fnmol.2022.965697 (PMC9589483; doi:10.3389/fnmol.2022.965697)
Supplement: Supplementary file 1 [file Data_Sheet_1.docx]

1. LC/MS-based metabolomic analysis and comparative analysis

Detailed descriptions of the liquid chromatography mass spectrometry (LC–MS) approaches and procedures used for metabolomic analysis in previous study [1]. Metabolites extracts were prepared from mice olfactory bulb. First, homogenization of olfactory bulb was performed on ice with 200 μL of prechilled water for 60s. Then, the samples were added with 800 μL of prechilled mixturescontaining 400 μL of methanol and 400 μL of water and homogenized on ice for 60 s. Second, the homogenate was sonicated on ice (30 min/once, twice). Then, proteins were removed by precipitation at -20 ◦C for 1 h and centrifugation at 14,500 g at 4 ◦C for 20 min. Finally, supernatant was dried in a vacuum centrifuge and redissolved in 100 μL solution containing 50 μL acetonitrile and 50 μL water. Quality Control samples were generated by pooling 10 μL of each biological sample.

The subsequent procedures included chromatographic separation on an Agilent 1290 Infinity LC system (Agilent Technologies, Santa-Clara, California, USA) and quadrupole time-off light mass spectrometry on an AB SCIEX Triple TOF 6600 System (AB SCIEX, Framingham, MA, USA). The LC-MS metabolic profiles were converted into the NetCDF format using TagFinder [2]. The normalized data set was imported into SIMCA-P 13.0 (Umetrics, Umea, Sweden) for multivariate statistical

analyses. Principal component analysis (PCA) was used to observe the distributions of all samples. To maximize class discrimination, the data further underwent orthogonal partial least-squares discriminant analysis (OPLS-DA), which was validated by a 200-iteration permutation test to avoid model overfitting. Metabolites with a VIP (variable importance in the projection) value > 1 in the OPLS-DA model and *P* < 0.05 in the

Student’s t-test were recognized as differentially expressed metabolites [3,4]. A heatmap was constructed using MetaboAnalyst 5.0. To explore the potential mechanisms contributing to encephalic region differences in MD, MetaboAnalyst 5.0 [5] was used to perform hierarchical clustering heatmap and metabolic pathway analysis.

**References:**

[1] L Fan, LN Yang, XM Li, T Teng, YJ Xiang, X Liu, et al. Proteomic and metabolomic characterization of amygdala in chronic social defeat stress rats. Behavioural Brain Research 412 (2021) 113407. https://pubmed.ncbi.nlm.nih. gov/ 34111472.

[2] A. Luedemann, K. Strassburg, A. Erban, J. Kopka, TagFinder for the quantitative

analysis of gas chromatography–mass spectrometry (GC-MS)-based metabolite

profiling experiments, Bioinformatics 24 (5) (2008) 732–737, https://doi.org/

10.1093/bioinformatics/btn023.

[3] T. Teng, C.A. Shively, X. Li, X. Jiang, G.N. Neigh, B. Yin, Y. Zhang, L. Fan, Y. Xiang, M. Wang, X. Liu, M. Qin, X. Zhou, P. Xie, Chronic unpredictable mild stress

produces depressive-like behavior, hypercortisolemia, and metabolic dysfunction

in adolescent cynomolgus monkeys, Transl. Psychiatry 11 (1) (2021) 9, https://doi.

org/10.1038/s41398-020-01132-6.

[4] M. Liu, H. Xie, Y. Ma, H. Li, C. Li, L. Chen, B. Jiang, B. Nian, T. Guo, Z. Zhang,

W. Jiao, Q. Liu, T. Ling, M. Zhao, High performance liquid chromatography and

metabolomics analysis of tannase metabolism of gallic acid and gallates in tea

leaves, J. Agric. Food Chem. 68 (17) (2020) 4946–4954, https://doi.org/10.1021/

acs.jafc.0c00513.

[5] J. Chong, D.S. Wishart, J. Xia, Using MetaboAnalyst 4.0 for comprehensive and

integrative metabolomics data analysis, Curr. Protoc. Bioinformatics 68 (1) (2019)

e86, https://doi.org/10.1002/cpbi.86.
